# Impact of Female Sex and Mild Cortisol Secretion on Coagulation Profile in Adrenal Incidentalomas

**DOI:** 10.1210/jendso/bvae215

**Published:** 2024-12-12

**Authors:** Ilaria Bonaventura, Marianna Minnetti, Davide Ferrari, Valeria Hasenmajer, Alessandra Tomaselli, Dario De Alcubierre, Andrea Lenzi, Riccardo Pofi, Andrea M Isidori

**Affiliations:** Department of Experimental Medicine, Sapienza University of Rome, Rome 00161, Italy; Department of Experimental Medicine, Sapienza University of Rome, Rome 00161, Italy; Department of Experimental Medicine, Sapienza University of Rome, Rome 00161, Italy; Department of Experimental Medicine, Sapienza University of Rome, Rome 00161, Italy; Department of Experimental Medicine, Sapienza University of Rome, Rome 00161, Italy; Department of Experimental Medicine, Sapienza University of Rome, Rome 00161, Italy; Department of Experimental Medicine, Sapienza University of Rome, Rome 00161, Italy; UNESCO Chair On “Urban Health—Education and Research for Improved Health and Wellbeing in the Cities,” Sapienza University of Rome, Rome 00161, Italy; Oxford Centre for Diabetes, Endocrinology and Metabolism, NIHR Oxford Biomedical Research Centre, University of Oxford, Churchill Hospital, Oxford OX3 7LJ, UK; Department of Experimental Medicine, Sapienza University of Rome, Rome 00161, Italy; Centre for Rare Diseases (Endo-ERN accredited), Policlinico Umberto I, Rome 00161, Italy

**Keywords:** adrenal incidentalomas, mild autonomous cortisol secretion, hypercoagulability, venous thromboembolism, coagulation factors, gender medicine

## Abstract

**Context:**

Studies describing the coagulation profile in adrenal adenomas still need to be added.

**Objective:**

We explored how sex and mild autonomous cortisol secretion (MACS) affect coagulation parameters in patients with adrenal adenomas.

**Design:**

Cross-sectional study.

**Methods:**

From January 2019 until April 2023, participants in the Impact of Adrenal IncidenTalomas and Possible Autonomous Cortisol Secretion on Cardiovascular and Metabolic Alterations trial (NCT04127552) diagnosed with adrenal adenoma were categorised according to the 1 mg overnight dexamethasone-suppression test (1 mg-DST). Coagulation parameters were evaluated, and two-way ANOVA was used to elucidate the cortisol-by-sex interaction.

**Results:**

Of 153 patients screened, 90 were enrolled (62.2% female, mean age 62 ± 10 years): 41 with non-functioning adrenal tumour (1 mg-DST ≤ 1.8 µg/dL), and 49 with a MACS (1 mg-DST > 1.8 µg/dL). Platelet counts were higher in the MACS group (*P* = .01). Regression analysis identified female sex (B = 36.603, *P* = .011), 1mg-DST (B = 0.238, *P* = .042), and younger age (B = −1.452, *P* = .038) as independent predictors for elevated platelet count. In patients with MACS, women exhibited higher levels of procoagulant factors fibrinogen (*P* = .004) and factor VIII (*P* < .001), and coagulation inhibitors protein C (*P* = .003) and antithrombin III (*P* = .005) than males. No differences were observed in the non-functioning adrenal tumour group, providing a cortisol-by-sex interaction regarding fibrinogen (*P* = .047), factor VIII (*P* = .046), and protein C (*P* = .028).

**Conclusion:**

Our findings revealed a worse coagulation profile in women with MACS, underscoring the need for a sex-specific approach in clinical practice to manage thrombotic risks effectively. Dedicated prospective studies are needed to validate and integrate these findings into clinical strategies for thromboprophylaxis.

Nowadays adrenal incidentalomas are increasingly detected due to the widespread use of high-resolution imaging techniques, with a prevalence of 5% to 7% in adults, increasing with age [1-6]. The majority of these lesions are benign adenomas originating from the adrenal cortex. Adrenal adenomas are classified based on morning serum cortisol levels following 1 mg overnight dexamethasone-suppression test (1 mg-DST) and, remarkably, up to 50% exhibit mild autonomous cortisol secretion (MACS), defined as 1 mg-DST > 1.8 µg/dL (50 nmol/L) [7, 8]. While often asymptomatic, MACS is linked to an elevated risk of morbidity, mortality, and, importantly, cardiovascular complications and thromboembolic events [9-18]. The latter association is especially pronounced in the context of overt hypercortisolism, where patients with Cushing's syndrome (CS) exhibit a significantly heightened risk of venous thromboembolic events (VTE) due to hypercoagulability [19-27]. However, despite that a recent study revealed a nonnegligible prevalence of thromboembolic events among individuals with MACS, the impact of MACS on coagulation parameters has been relatively underexplored [10]. The interplay between sex differences and cardiovascular health outcomes is increasingly recognized as a pivotal area of medical research [28, 29], particularly regarding hypercoagulability and thrombotic events [28, 30-32], highlighting the necessity for gender medicine to tailor personalized, effective treatments [33]. Notably, women, especially those under 65 with MACS, face worse outcomes compared to men, underscoring a complex interplay of sex, hormonal levels, and hypercortisolism [10]. Nevertheless, the specific impact of mild hypercortisolism on coagulation parameters and the extent to which sex-specific differences contribute are yet to be fully clarified. This underscores the critical need to further deepen the mechanisms driving these sex differences to inform risk assessment and personalized management strategies in patients with mild hypercortisolism.

This study aims to bridge this knowledge gap by examining coagulation parameters in asymptomatic patients with adrenal adenoma, focusing on the contributions of MACS and investigating potential sex-specific differences.

## Material and Methods

### Patient Selection

This is a prospective observational study (Impact of Adrenal IncidenTalomas and Possible Autonomous Cortisol Secretion on Cardiovascular and Metabolic Alterations, NCT04127552) on consecutive patients aged 18 to 80 years diagnosed with adrenal incidentaloma and referred to the endocrine outpatient clinic of the Department of Experimental Medicine, Policlinico Umberto I, Sapienza University of Rome. Only patients with adrenal adenoma were included in the study. Exclusion criteria included a confirmed diagnosis of overt CS, defined according to the guidelines as the presence of hypercortisolism (evaluated by the 1 mg-DST, 24 hours urinary free cortisol (UFC), and/or late-night salivary cortisol) along with specific clinical signs of cortisol excess (eg, easy bruising, facial plethora, and proximal myopathy) [34, 35]. Patients with a diagnosis of adrenal masses other than nonaldosterone-secreting adrenocortical adenomas, such as primary aldosteronism, pheochromocytoma, adrenocortical carcinoma, adrenal metastasis, myelolipoma, congenital adrenal hyperplasia, and other rare adrenal diseases were excluded, along with patients using drugs or affected by diseases known to impact on corticosteroid metabolism or cortisol secretion (ie, glucocorticoids, hormone replacement therapy or hormonal contraceptive, thyrotoxicosis, bowel diseases, chronic renal failure, chronic hepatic disease, depression, alcoholism, eating disorders, rheumatologic and hematological diseases). Postmenopausal women receiving any form of estrogen replacement therapy were excluded from the study. Moreover, patients taking anticoagulants with a history of thrombosis or myocardial infarction and an active malignancy were also excluded from the study. In accordance with the most recent European Society of Endocrinology clinical practice guidelines on the management of adrenal incidentalomas, in collaboration with the European Network for the Study of Adrenal Tumors [10], all patients were stratified for cortisol secretion as follows: patients with 1 mg-DST above 1.8 µg/dL (>50 nmol/L) were labeled as MACS, whereas those 1 mg-DST ≤ 1.8 µg/dL (≤50 nmol/L) were classified as non-functioning adrenal tumors (NFATs). In all patients with MACS, the diagnosis of overt CS was excluded based on physical examination and at least 2 repeated UFC measurements. Therefore, UFC values were calculated as the mean of at least 2 independent measurements.

Anthropometric measurements included body mass index (BMI), calculated by dividing weight (in kg) by squared height (in m^2^). Arterial hypertension was defined by systolic blood pressure ≥ 140 mmHg and/or diastolic blood pressure ≥ 90 mmHg on repeated evaluations and/or by the reported use of antihypertensive medication [36]. Type 2 diabetes mellitus, impaired fasting glucose, and impaired glucose tolerance were diagnosed according to fasting glucose or hemoglobin A1c as per European Society of Cardiology/European Association for the Study of Diabetes guidelines or reported use of antidiabetic drugs [37]; insulin resistance (IR) was defined by the Homeostasis Model Assessment of Insulin Resistance ≥ 2.5 [38]. Dyslipidaemia was diagnosed according to lipid profile as per European Society of Cardiology/European Association for the Study of Diabetes guidelines or reported use of hypolipidemic drugs [37]. As smoking is a well-established thromboembolic risk factor, with an immediate increase in the risk of thrombosis for current smokers and a gradual decrease over time for former smokers, we have taken these variables into account and defined “current smoker” as an individual who was smoking any tobacco at the time of the study and “former smoker” as an individual who had quit smoking although they had smoked in the past [39-41]. Adrenal lesions were discovered by computed tomography or magnetic resonance imaging, and all of them were larger than 10 mm and displayed magnetic resonance or computed tomographyCT features consistent with benign adenoma. When bilateral adenomas were found, the diameter of the largest adenoma was considered. This study was approved by the Local Ethics Committee of Sapienza University in Rome (reference number 5279) and was conducted in accordance with the Declaration of Helsinki (1964) and its subsequent amendments. All enrolled patients provided their written informed consent to participate in the study.

### Laboratory Analyses

All biochemical tests were performed locally at the study center. Serum cortisol was measured by radioimmunoassay, using Beckman Coulter reagents (ref. IM1841, RRID: AB_2894408). The measurement range (from analytical sensitivity to the highest calibrator) is 8.60 to 2000 nM. The high specificity of the assay was confirmed by extremely low or undetectable cross reactivity against other naturally occurring steroids (aldosterone, corticosterone, cortisone, 11-desoxycortisol, progesterone, etc.) or therapeutic drugs that may be present in patient samples (eg, prednisolone, prednisone, spironolactone). Complete blood count, coagulation markers factor V (FV), factor VII (FVII), factor VIII (FVIII), fibrinogen, prothrombin time (PT), international normalization ratio (INR), activated partial thromboplastin time (aPTT) and coagulation inhibitors such as antithrombin III (ATIII), protein C anticoagulant (protein C), and protein S were performed. PT, aPTT, and INR were measured by standard methods. Standard 1-stage clotting assays using a specific factor-deficient plasma as substrate were used to determine FV, FVII, and FVIII (Siemens Medical Solutions Diagnostics). AT III and protein C activity were quantified by chromogenic determination (Siemens Medical Solutions Diagnostics). Protein S activity was quantified with a clotting assay (Siemens Medical Solutions Diagnostics).

### Statistical Analysis

Distribution of continuous variables was assessed with the Shapiro–Wilk test; linearity was established by visual inspection of a scatterplot. Categorical variables are expressed as percentage and frequency; continuous variables are reported as mean and SD or median and interquartile range (IQR, 25th-75th percentile) as appropriate per distribution of data. For group comparisons unpaired Student's *t*-test, Mann–Whitney, χ^2^, or Fisher's exact test were used as appropriate. A backward stepwise predictor selection was applied to the linear regression analysis, starting with a full model and gradually eliminating any predictor that was not significant to the model. Multiple regression analysis was run to test the effects of variables selected based on the known association with increased VTE [42] (1 mg-DST, sex, BMI, age, smoking habits) on the likelihood of predicting coagulation parameters showing significant group differences. Multicollinearity among the predictor variables was identified by assessing the values of tolerance and variance inflation factor. Two-way ANOVA was conducted to assess the main effects of the 2 independent variables (cortisol secretion, based on 1 mg-DST, and sex) and their interaction effect on the dependent variable (coagulation parameters) and was interpreted as significant at *P*-value <.05 and possible for .05 ≤ *P-*value <.10. Post hoc analyses (Bonferroni correction) were performed to compare specific levels of the independent variables, when adequate. A log transformation or reciprocal transformation was used for skewed data, as appropriate. Outliers, constituting less than 2% of the sample, were detected using the IQR method in the boxplot and removed to ensure more accurate ANOVA results. Homogeneity of variances was assessed by Levene's test. Statistical analyses were performed using SPSS, version 29 (IBM, Chicago, IL) and GraphPad Prism 10.0 software package (GraphPad Software, La Jolla, CA).

## Results

### Characteristics of the Whole Cohort

**Figure 1.**
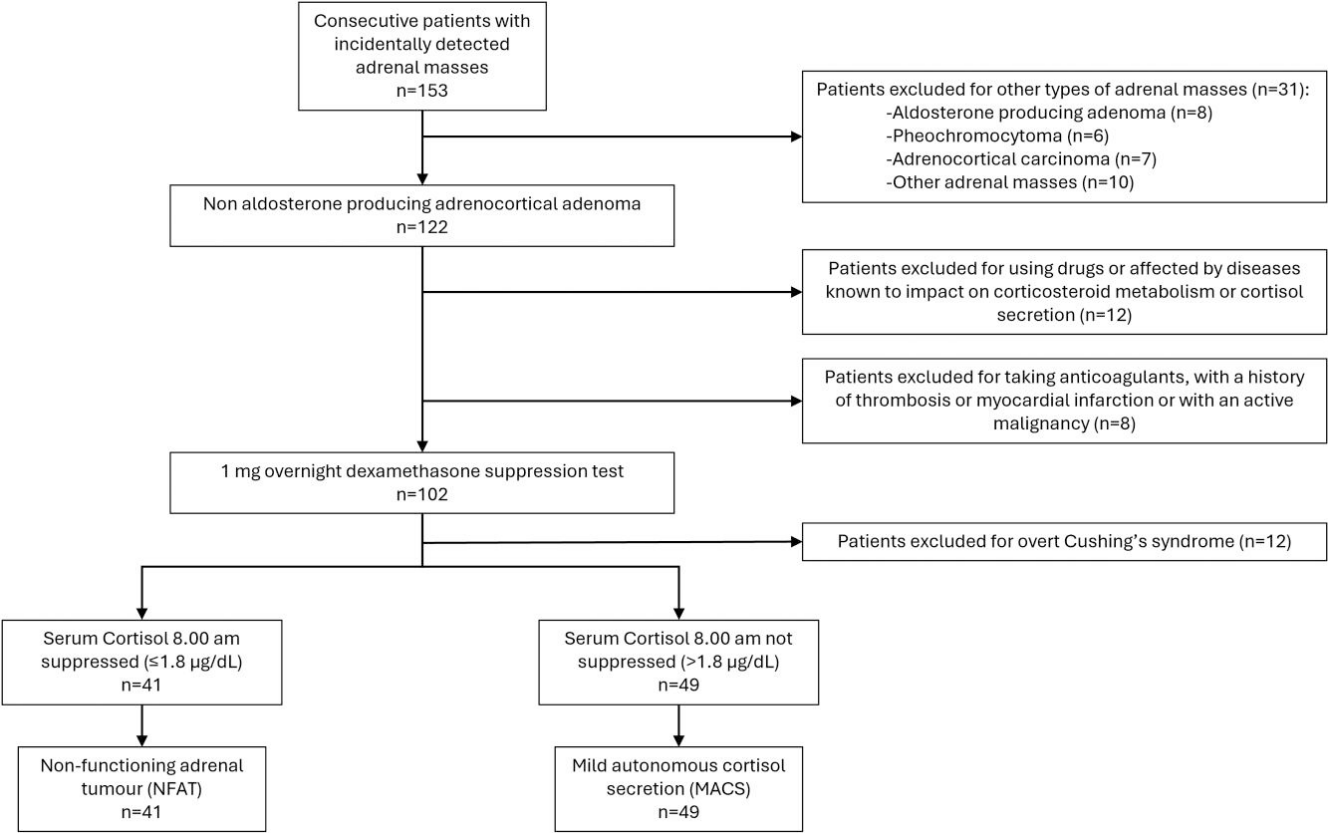
Study flowchart.

Between September 2019 and April 2023, out of 153 patients with adrenal incidentaloma referred to our department, 90 qualified for the coagulation study inclusion criteria (Fig. 1). The mean age of the participants was 62 ± 10 years, with a median BMI of 25.7 kg/m^2^ (IQR 23.6-29.9). In our cohort, all patients indicated that their gender corresponded to their sex registered at birth; all were Caucasian. Among these, 56 (62.2%) were female, aged between 49 and 71 years; all were postmenopausal with a median estradiol 10 pg/mL (IQR 10-17) and a mean FSH 51.1 ± 8.7 mIU/mL, and none were on hormone replacement therapy. All males were eugonadal with mean testosterone levels of 18.7 ± 1.5 nmol/L. According to BMI, 36.7% presented with overweight and 23.3% presented with obesity. The overall prevalence of cardiovascular comorbidities was 60% for arterial hypertension and 59% for dyslipidemia. Impaired glucose metabolism was found in 47 patients (52%), including 8.9% with type 2 diabetes mellitus and 43.3% with either impaired fasting glucose, impaired glucose tolerance, or insulin resistance. The maximum diameter of the adrenal lesion was 20.0 mm (IQR 14.0-31.0). According to the number of adrenal lesions, 21 (23.3%) had bilateral adenomas, of which 6 (28.6%) had more than 2 nodules on at least 1 side. A summary of characteristics of the whole cohort is shown in Table 1.

**Table 1.** Characteristics and comorbidities of the whole cohort and after stratifying for 1 mg dexamethasone suppression test

	All (n = 90)	MACS (n = 49)	NFAT (n = 41)	*P*-value
Age	62 ± 10	63 ± 10	61 ± 10	.197
BMI	25.7 [23.6-29.9]	27.1 [23.5-31.1]	24.9 [23.6-28.2]	.214
Females (%)	56 (62.2)	33 (67.3)	23 (56.1)	.339
Adrenal lesion size (mm)	20.0 [14.0-31.0]	27.0 [19.0-37.5]	16.5 [12.0-21.5]	**<.001**
Bilateral lesions (%)	21 (23.3)	16 (32.7)	5 (12.2)	**.043**
1-mg DST (µg/dL)	2.0 [1.4-3.1]	3.0 [2.5-4.2]	1.2 [1.1-1.6]	**<.001**
UFC (×ULN)	0.62 [0.46-0.91]	0.60 [0.40-0.70]	0.55 [0.46-0.66]	.675
ACTH (pg/mL)	18.9 [13.1-24.2]	16.6 [11.2-20.9]	20.3 [15.6-34.5]	**.009**
SBP (mmHg)	131 ± 16	130 ± 16	132 ± 16	.580
DBP (mmHg)	80 ± 9	78 ± 9	81 ± 10	.256
Sodium (mmol/L)	142 ± 2	142 ± 2	142 ± 2	.834
Potassium (mmol/L)	4.3 ± 0.4	4.3 ± 0.4	4.3 ± 0.4	.736
Current smokers (%)	33 (36.7)	20 (40.8)	13 (31.7)	.510
Former smokers (%)	9 (10)	5 (10.2)	4 (9.8)	.975
Patients with overweight (%)	33 (36.7)	21 (42.9)	12 (29.3)	.212
Patients with obesity (%)	21 (23.3)	13 (26.5)	8 (19.5)	.470
Type 2 diabetes (%)	8 (8.9)	5 (10.2)	3 (7.3)	.657
IFG/IGT/IR (%)	39 (43.3)	24 (49.0)	15 (36.6)	.278
Arterial hypertension (%)	54 (60)	31 (63.3)	23 (56.1)	.580
Dyslipidemia (%)	53 (58.9)	31 (63.3)	22 (53.7)	.429

Continuous data are expressed as mean ± SD or median [interquartile range], as appropriate. Categorical variables are expressed as frequency (%). Significant *P*-values are highlighted in bold.

Abbreviations: 1 mg-DST, serum cortisol following 1 mg dexamethasone suppression test; BMI, body mass index; DBP, diastolic blood pressure; IFG, impaired fasting glucose; IGT, impaired glucose tolerance; IR, insulin resistance; MACS, mild autonomous cortisol secretion; NFAT, nonfunctioning adrenal tumor; SBP, systolic blood pressure; UFC, urinary free cortisol; ULN, upper limit of normal.

### Subgroup Analysis According to 1 mg-DST

**Figure 2.**
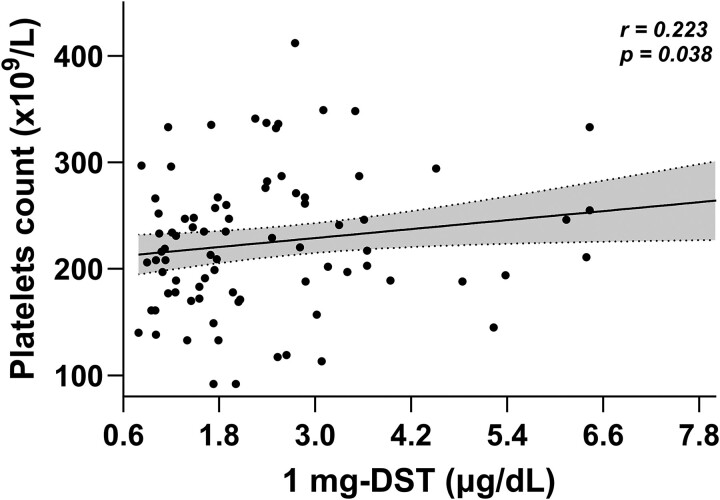
Univariate analysis for 1 mg-DST and platelets count in a cohort of 90 patients with adrenal adenomas. Grey areas between the dashed lines refer to the 95% confidence intervals of the regression lines. Abbreviation: 1 mg-DST, 1 mg dexamethasone suppression test.

Forty-one patients were classified as NFAT (45.5%) and 49 as MACS (54.5%). Both groups were comparable in terms of sex, age, BMI, and smoking habits (current/former smokers) distributions. There were no significant differences in the frequency of comorbidities between groups. Notably, MACS had larger adrenal lesions (27.0 mm, IQR 19.0-37.5) compared to NFAT (16.5 mm, IQR 12.0-21.5; *P* < .001), lower ACTH levels (16.6 pg/mL, IQR 11.2-20.9 *vs* 20.3 pg/mL IQR 15.6-34.5, *P* = .009), and a higher prevalence of bilateral lesions compared to NFAT (32.7% vs 12.2%, *P* = .043), as shown in Table 1. UFC levels were below the upper limit of normal (ULN) in both groups, with no significant differences observed (0.60 × ULN [0.40-0.70] vs 0.55 × ULN [0.46-0.66], *P* = .675). Additionally, no correlation between age and the 1 mg-DST was observed (r = 0.144, *P* = .182). No differences in coagulation markers and inhibitors were found between MACS and NFAT, except for the platelets count, which was higher in the MACS compared to the NFAT group (242 ± 72 × 10^9^/L vs 209 ± 54 × 10^9^/L, *P* = .01), as shown in Table 2. Additionally, a univariate analysis across the whole cohort identified a positive relationship between 1 mg-DST and platelet count (r = 0.223; *P* = .038), as shown in Fig. 2.

**Table 2.** Coagulation parameters of patients enrolled in the study, divided according to 1 mg dexamethasone suppression test

	MACS (n = 49)	NFAT (n = 41)	*P*-value
Platelets (×10^9^/L)	242 ± 72	209 ± 54	**.010**
Fibrinogen (g/L)	3.4 [2.7-3.9]	3.2 [2.9-3.8]	.530
INR	0.98 ± 0.06	0.97 ± 0.06	.340
PT (%)	114.0 ± 9.7	110.1 ± 10.5	.094
aPTT (sec)	28.8 [27.0-31.3]	29.1 [27.0-31.7]	.720
FV (%)	123 ± 32	130 ± 26	.250
FVII (%)	123 [96-148]	119 [102-150]	.670
FVIII (%)	133 [115-152]	146 [110-156]	.560
AT III (%)	95 [87-108]	99 [91-113]	.190
Protein C (%)	119 [96-150]	117 [106-144]	.730
Protein S (%)	96 ± 21	98 ± 18	.730

Continuous data are expressed as mean ± SD or median [interquartile range], as appropriate. Significant *P*-values are highlighted in bold.

Abbreviations: 1 mg-DST, serum cortisol following 1 mg dexamethasone suppression test; aPTT, activated partial thromboplastin time; AT III, antithrombin III; FV, factor V; FVII, factor VII; FVIII, factor VIII; INR, international normalization ratio; MACS, mild autonomous cortisol secretion; NFAT, nonfunctioning adrenal tumor; protein C, protein C anticoagulant; PT, prothrombin time.

To ascertain if a set of clinically and biochemically important parameters could independently predict total platelet count, a backward linear regression analysis was performed. The analysis included factors considered potentially associated with increased risk for VTE (ie, 1 mg-DST, sex, BMI, age, smoking habits) [42]. The model was significant (r^2^ = 0.169, *P* = .009) revealing that female sex had the most substantial influence (*B* = 36.603, *P* = .011) on total platelet count. Additionally, a higher 1 mg-DST (*B* = 0.238, *P* = .042) and a younger age (*B* = −1.452, *P* = .038) were also identified as significant, albeit less influential, predictors. Notably, smoking habits or BMI did not appear to contribute to platelet total count. All coefficients and complete results are reported in Table 3. No differences in coagulation parameters were observed between patients with unilateral lesion and those with bilateral lesions.

**Table 3.** Backward stepwise predictor selection applied to multiple regression analysis in 90 patients with adrenal adenoma

	Dependent: Platelet count			
Independent	B	95% CI lower bound	95% CI upper bound	*P*-value
Model 1				
Age	−1.326	−2.726	0.074	.063
Female sex	37.312	8.183	66.442	.**013**
BMI	1.132	−1.757	4.021	.438
Smoking habit (current, former, and nonsmokers)	4.098	−10.931	19.127	.589
1 mg-DST	0.223	−0.017	0.463	.068
Model 2				
Age	−1.362	−2.756	0.032	.055
Female sex	36.508	8.605	64.410	.**011**
BMI	1.086	−1.756	3.927	.449
1 mg-DST	0.238	0.007	0.468	.**043**
Model 3				
Age	−1.452	−2.822	−0.082	.**038**
Female sex	36.603	8.778	64.428	.**011**
1 mg-DST	0.238	0.008	0.468	.**042**

The dependent variable was total platelets count. The independent variables were age, sex, BMI, serum cortisol following 1 mg-DST, smoking habits (current, former, and no smokers). Significant *P*-values are highlighted in bold (r^2^ = 0.169, *P* = .009).

Abbreviations: 1 mg-DST, serum cortisol following 1 mg dexamethasone suppression test; BMI, body mass index; CI, confidence interval.

### Subgroup Analysis According to sex

We therefore sought to analyze sex differences across the entire cohort. Despite both groups being comparable in terms of age, BMI, smoking habits (current/former smokers) distributions, prevalence of MACS, and bilateral adenomas, females displayed a less favorable coagulation profile than males, characterized by a higher platelets count (242 ± 59 ×10^9^/L vs 202 ± 71 × 10^9^/L, *P* = .003), fibrinogen (3.4 g/L [IQR 3.0-3.9] vs 3.0 g/L [IQR 2.7-3.7], *P* = .043), FV (132 ± 30% vs 119 ± 25%, *P* = .044), FVII (130% [IQR 104-174] vs 107% [IQR 93-126], *P* = .002), and ATIII (104% [IQR 93-116] vs 93% [IQR 87-100], *P* = .006), and lower INR (0.96 ± 0.05 vs 0.99 ± 0.06, *P* = .023) and aPTT (28.4 second [IQR 26.5-30.5] vs 30.4 second [IQR 27.9-32.4], *P* = .017) (Table 4).

**Table 4.** Characteristics and coagulation parameters of patients enrolled in the study, divided according to sex

	Female (n = 56)	Male (n = 34)	*P*-value
Age	63 ± 9	61 ± 12	.218
BMI	25.7 [23.4-29.7]	25.7 [23.7-31.2]	.726
Current smokers (%)	19 (33.9)	14 (41.2)	.274
Former smokers (%)	4 (7.1)	5 (14.7)	.366
MACS (%)	33 (58.9)	16 (47.0)	.511
Bilateral lesions (%)	13 (23.2)	8 (23.5)	.815
Platelets (×10^9^/L)	242 ± 59	202 ± 71	**.003**
Fibrinogen (g/L)	3.4 [3.0-3.9]	3.0 [2.7-3.7]	**.043**
PT (%)	113.3 ± 10.2	110.2 ± 10.2	.100
INR	0.96 ± 0.05	0.99 ± 0.06	**.023**
aPTT (sec)	28.4 [26.5-30.5]	30.4 [27.9-32.4]	**.017**
FV (%)	132 ± 30	119 ± 25	**.044**
FVII (%)	130 [104-174]	107 [93-126]	**.002**
FVIII (%)	146 [122-159]	126 [101-152]	.052
AT III (%)	104 [93-116]	93 [87-100]	**.006**
Protein C (%)	137 [108-149]	112 [102-132]	.083
Protein S (%)	97 ± 21	98 ± 17	.895

Continuous data are expressed as mean ± SD or median [interquartile range], as appropriate. Significant *P*-values are highlighted in bold.

Abbreviations: aPTT, activated partial thromboplastin time; AT III, antithrombin III; FV, factor V; FVII, factor VII; FVIII, factor VIII; INR, international normalization ratio; protein C, protein C anticoagulant; PT, prothrombin time.

Noteworthy, a further stratification by sex within the MACS and NFAT groups revealed significant differences in the MACS group alone, where females confirmed a less favorable coagulation profile than males, as detailed in Supplementary Table S1 [43]. Conversely, no sex differences in the coagulation profile were observed in the NFAT group.

### Interaction of Cortisol Secretion and sex on the Coagulation Profile of Adrenal Adenomas

**Figure 3.**
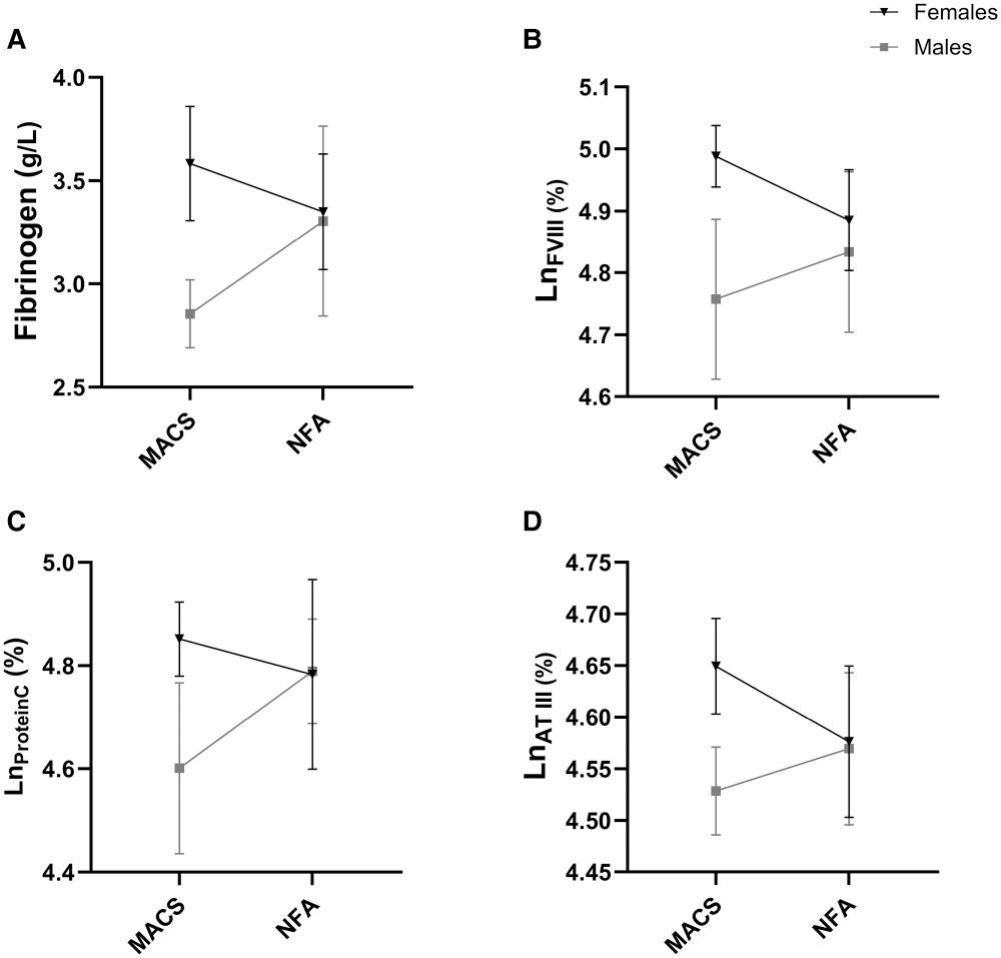
Impact of sex (female vs male) and cortisol secretion (MACS vs NFAT) and their interaction on coagulation markers such as fibrinogen (A), FVIII (B), and coagulation inhibitors such as protein C (C) and AT III (D). Data are expressed as means ± SD and analyzed by ANOVA test. Abbreviations: AT III, antithrombin III; FVIII, factor VIII; MACS, mild autonomous cortisol secretion; NFAT, nonfunctioning adrenal tumor; protein C, protein C anticoagulant.

To elucidate the potential interaction between the effects of cortisol secretion excess, based on 1 mg-DST, and sex (cortisol-by-sex interaction) on coagulation parameters, a 2-way ANOVA was performed. As opposed to patients with NFAT (Fig. 3 and Table 5), the analysis showed that, in patients with MACS, women exhibited higher levels of both procoagulant factors, including fibrinogen [females 3.58 ± 0.76 g/L vs males 2.85 ± 0.24 g/L; Δ_Sex_ 0.73 g/L, 95% confidence interval (CI) 0.24 to 1.22; *P* = .004] and FVIII (females 147 ± 1% vs males 117 ± 1%; Δ_Sex_ 1.26%, 95% CI 1.11 to 1.42; *P* < .001) and coagulation inhibitors, such as protein C (females 128 ± 1% vs males 99 ± 1%; Δ_Sex_ 1.28%, 95% CI 1.09 to 1.50; *P* = .003) and ATIII (females 105 ± 1% vs males 93 ± 1%; Δ_Sex_ 1.1%, 95% CI 1.0 to 1.2; *P* = .005), compared to males (Fig. 3 and Table 5). This resulted in a cortisol-by-sex interaction on fibrinogen (*P* = .047), FVIII (*P* = .046), and protein C (*P* = .028) and a possible interaction for ATIII, which approached statistical significance (*P* = .060). Other parameters, including platelets count, PT, INR, aPTT, FV, FVII, and protein S did not show significant differences (Supplementary Fig. S1 [44] and Table 5).

**Table 5.** Change in coagulation parameters

		Δ Sex (female-male)	Between-group *P-*value	Cortisol-by-sex interaction *P*-value
Platelets (×10^9^/L)	MACS	23.9 (−12.4; 60.1)	.193	.894
	NFAT	20.3 (−19.7; 60.2)	.316	
Fibrinogen (g/L)	MACS	0.73 (0.24; 1.22)	.**004**	.**047**
	NFAT	0.04 (−0.42; 0.51)	.857	
PT (%)	MACS	3.48 (−3.62; 10.58)	.332	.687
	NFAT	1.49 (−5.30; 8.27)	.664	
INR	MACS	−0.02 (−0.06; 0.01)	.206	.666
	NFAT	−0.03 (−0.07; 0.03)	.069	
aPTT (sec)	MACS	−2.72 (−5.05; 0.40)	.**022**	.440
	NFAT	−1.44 (−3.76; 0.87)	.218	
FV (%)	MACS	11.2 (−6.4; 28.7)	.210	.851
	NFAT	13.6 (−4.78; 31.93)	.145	
FVII (%)	MACS	1.22 (1.03; 1.44)	.**019**	.945
	NFAT	1.23 (1.03; 1.46)	.**020**	
FVIII (%)	MACS	1.26 (1.11; 1.42)	**<**.**001**	.**046**
	NFAT	1.05 (0.93; 1.19)	.408	
ATIII (%)	MACS	1.1 (1.0; 1.2)	.**005**	.060
	NFAT	1.0 (0.9; 1.1)	.921	
Protein C (%)	MACS	1.28 (1.09; 1.50)	.**003**	.**028**
	NFAT	0.99 (0.86; 1.16)	.928	
Protein S (%)	MACS	1.02 (0.86; 1.20)	.814	.485
	NFAT	0.94 (0.79; 1.12)	.461	

ANOVA, values are expressed as mean (lower upper limit of 95% confidence interval). Significant *P*-values are highlighted in bold.

Abbreviations: 1 mg-DST, serum cortisol following 1 mg dexamethasone suppression test; aPTT, activated partial thromboplastin time; AT III, antithrombin III; FV, factor V; FVII, factor VII; FVIII, factor VIII; INR, international normalization ratio; MACS, mild autonomous cortisol secretion; NFAT, nonfunctioning adrenal tumor; protein C, protein C anticoagulant; PT, prothrombin time.

## Discussion

This study investigates coagulation parameters in a homogeneous cohort of patients with adrenal adenomas, exploring the influence of sex and cortisol levels on hemostasis. While the presence of mild hypercortisolism on its own may not markedly alter the coagulation system, a distinct sex-related difference is observed within the MACS group, with females presenting a worse profile compared to their male counterparts.

Previous research has established a link between hypercortisolism and an enhanced coagulation system, particularly evident in CS, which typically involves an alteration of the intrinsic coagulation pathway, in particular an increase in FVIII, and increased levels of coagulation inhibitors (ie, ATIII, protein C, and protein S) as a compensatory response [22]. In line with this, the most recent consensus on the diagnosis and management of CS suggests that clinicians and surgeons should be aware of the altered coagulation profile in patients with CS for up to 1 year following surgical remission and should consider administering anticoagulation therapy on a case-by-case basis [45].

A recent meta-analysis on more than 7,000 subjects with CS revealed a substantially increased risk (OR 18) of VTE compared to the general population. As expected, the majority of the subjects were female (78%), yet no direct link was found between the severity of hypercortisolism and VTE incidence or coagulation profiles. Notably, this analysis did not explore differences in outcomes based on the interaction between sex and cortisol levels [23]. On the contrary, results from the ERCUSYN study indicated that both male sex [hazards ratio (HR) 2.11] and higher UFC levels (HR 1.06) significantly increased the risk of VTE occurrence. However, data on UFC levels in patients with VTE were only available in 50% of patients, and, importantly, men exhibited higher UFC levels than women [19]. Nevertheless, nowadays it is recognized that MACS and CS are distinct entities, with the transition from MACS to CS a rare occurrence [7, 46-49]. However, both conditions share increased cardiovascular and metabolic risk compared to NFAT, suggesting that even a mild cortisol excess might contribute to these risks.

In our cohort, the significant difference in platelet counts between MACS and NFAT groups, despite remaining within the normal range, points toward a potential elevated thrombotic risk associated with the former, albeit this interpretation warrants dedicated studies to confirm any clinically significant implications. However, the absence of significant differences in other coagulation markers implies that mild hypercortisolism alone may not drastically alter coagulation function. Our regression analysis, which considered variables typically associated with hypercoagulability, such as age, sex, BMI, smoking habits, and 1 mg-DST, specifically highlighted that younger (albeit postmenopausal) women with MACS presented with higher platelet counts, independent of typical risk factors like obesity or smoking habits. Several studies have reported a reduction in platelet counts with aging [50, 51]. Our analysis confirms these findings, showing that for every year of decrease in age, there was an average increase of 1.5 × 10^9^/L in platelet count. Additionally, each 10 nmol/L (∼0.4 µg/dL) increase in 1 mg-DST was associated with a rise of 2.5 × 10^9^/L in platelet count. Notably, females had approximately 40 × 10^9^/L more platelets than their male counterparts, suggesting that female sex may be the most significant factor influencing platelet count in our study. Our results align with a retrospective study on over 300 patients with adrenal adenomas including NFA, MACS, and CS that found that women had higher platelet counts compared to men. However, the authors did not observe any difference across the spectrum of cortisol secretion [52]. Importantly, in this study, the groups differed in terms of age, with patients diagnosed with CS being on average 20 years younger than those with MACS and NFAT. Unfortunately, the analysis was not corrected by age [52]. Therefore, our finding underscores the nuanced relationship between cortisol levels and coagulation, suggesting that specific demographic and clinical features, including sex, age, and mild cortisol secretion, might contribute to an adverse coagulation profile. This complexity suggests the need for additional research to explore whether healthcare providers should develop personalized treatment plans for patients with these specific risk factors, aiming for more precise and effective care.

Following the latest guidelines by the European Society of Endocrinology/European Network for the Study of Adrenal Tumors on adrenal incidentalomas management, interpreting the 1-mg DST results as a spectrum rather than a binary outcome emphasizes the nuanced impact of cortisol levels on thrombotic risk [7]. Our study reinforces the concept that the degree of hypercortisolism, rather than the mere diagnosis of MACS, should be considered an additional risk factor for an altered coagulation profile, especially in women. This highlights the importance of thorough thrombotic risk assessments in patients with adrenal adenoma, and especially females, eligible for adrenalectomy [53] to potentially guide the implementation of perioperative anticoagulation strategies aimed at minimizing surgical thrombotic complications.

Our results complement and potentially elucidate the observation from the NAPACA outcome study [10]. In this large retrospective study with a 7-year median follow-up duration, the authors confirmed cardiovascular comorbidities to be more frequently observed in patients with MACS than in NFAT. Notably, there was a nonnegligible proportion of venous thrombosis and pulmonary embolism, suggesting the presence of a prothrombotic risk in this population. Importantly, the study found that the increase of all-cause mortality and a high prevalence of associated cardiometabolic comorbidities in patients with MACS was sex- and age-dependent [10]: women younger than 65 years of age with MACS had the worst outcomes (HR 4.39) suggesting a sexual dysmorphism among adrenal adenomas [29].

Aligned with these findings, our study further enriches the discussion on sex disparities in patients with adrenal incidentaloma. Our analysis reveals a distinct coagulation profile in females with MACS, despite remaining within the normal range, marked by elevated levels of fibrinogen, FVIII, protein C, and ATIII, and their male counterparts underscoring the critical role of sex in modulating coagulative risks. Notably, such distinctions were not evident in the NFAT group, emphasizing the predominant influence of cortisol levels on coagulation dynamics and suggesting an intricate interplay between cortisol secretion and sex. Determining whether the observed alterations in coagulation patterns in our cohort translates into a clinically significant increase in the incidence of VTE requires confirmation through dedicated prospective studies. However, several reports demonstrated a correlation between increased plasma concentrations of both FVIII and fibrinogen with increase of VTE risk [54-58]. A case-control study involving more than 300 participants revealed that subjects with FVIII levels above 150% experienced a nearly 5-fold increase in the risk of VTE compared to those with normal levels. Additionally, the study estimated that each 1% increase in FVIII levels over the normal range raised the odds ratio for VTE by 1.9% [54]. This finding extends to the recurrence of VTE [56]. Applying these insights to our cohort, a 34% difference in FVIII levels between females and males in the MACS group (Supplementary Table S1 [43]) suggests a 65% heightened risk of VTE in women with MACS compared to their male counterparts. These findings highlight the clinical significance of our data and support a refined sex-specific approach in assessing and managing the coagulation risks in patients with adrenal incidentaloma. This could potentially guide the integration of these findings into clinical strategies for thromboprophylaxis.

Previous research has reported sex-related disparities in coagulation markers, often linking differences in platelet activity to sex hormones [59-61]. Estrogens have been shown to stimulate prostacyclin production, leading to higher nitric oxide levels and reduced platelet aggregation [60, 62]. However, our study exclusively included postmenopausal women, indicating that the differences observed in coagulation parameters between females with MACS and those with NFAT could stem from other complex sex-specific interplays between cortisol and coagulation factors, beyond the hormonal changes observed in menopause. The precise mechanisms underlying these observations remain to be fully elucidated, calling for additional studies.

Although our study provides valuable insights, it is important to acknowledge its limitations, including a relatively small sample size and the lack of a control group (patients without adrenal adenomas), which makes it challenging to provide definitive data. In this study, we evaluated the contribution of major general thromboembolic risk factors such as smoking, sex, and BMI, as well as population-specific factors like the degree of hypercortisolism. However, several studies highlight that lifestyle factors, particularly dietary habits, can also influence coagulation status [63-65]. Therefore, the lack of investigation into this aspect represents an additional limitation of the study. Moreover, individual variations in drug metabolism or biological response may affect responses to the 1 mg-DST, and thus, the unavailability of dexamethasone serum concentrations represents another limitation of our study [66]. Additionally, being a single time point evaluation, we are unable to capture changes over time and demonstrate if the observed relationships among variables are constant over time (ie, after adrenalectomy, medical treatment, active follow-up). Therefore, further prospective studies are necessary to confirm whether sex differences in coagulation profiles among patients with adrenal adenomas lead to an increased incidence of VTE. Identifying additional thromboembolic risk scores that account for these differences could be beneficial, as current risk assessment models used in clinical practice primarily apply to hospitalized patients, exhibiting weak predictive accuracy for VTE [67]. Despite these limitations, our study provides a detailed analysis of coagulation parameters in patients with adrenal adenoma, representing a pioneering exploration of the potential for sex-based differences in this context. This aspect carries clinical implications, as it opens the possibility of the inclusion of this condition among known risk factors for VTE (ie, smoking, hormone replacement therapy or hormonal contraceptive, etc). Our data suggest redefining antithrombotic prophylaxis with a sex-specific approach and considering MACS in females as an additional risk factor warranting intervention in specific settings (ie, long flights or periods of immobilization). This lays the groundwork for further studies aimed at understanding whether patients with adrenal adenomas may benefit from antithrombotic prophylaxis and whether it requires greater attention compared to the general population.

## Conclusions

While coagulation function in CS has been extensively investigated, there remains a paucity of data on the coagulation status in patients diagnosed with adrenal incidentaloma. Generally, patients with adrenal adenomas, encompassing both NFAT and MACS, do not exhibit clinically significant alterations in coagulation parameters. However, considering sex differences, females diagnosed with MACS exhibit a worse coagulation profile. Nowadays, the consideration of sex dimorphism should be a key factor in personalized management strategies in patients diagnosed with adrenal incidentaloma. Such an approach has the potential not only to minimize the rising healthcare costs associated with this condition but also to underscore the importance of incorporating sex as a crucial factor in the assessment and management of adrenal incidentalomas and potentially integrate these findings into clinical strategies for thromboprophylaxis. Further prospective studies involving larger cohorts are essential to thoroughly elucidate the relationship between hypercortisolism and hypercoagulability in patients diagnosed with adrenal incidentaloma.

## Data Availability

The datasets generated during and/or analyzed during the current study are available from the corresponding author on reasonable request.

## Abbreviations

1 mg-DST: 1 mg overnight dexamethasone-suppression test
aPTT: activated partial thromboplastin time
ATIII: antithrombin III
BMI: body mass index
CI: confidence interval
CS: Cushing's syndrome
FV: factor V
FVII: factor VII
FVIII: factor VIII
HR: hazards ratio
INR: international normalization ratio
IQR: interquartile range
MACS: mild autonomous cortisol secretion
NFAT: nonfunctioning adrenal tumor
protein C: protein C anticoagulant
PT: prothrombin time
UFC: urinary free cortisol
ULN: upper limit of normal
VTE: venous thromboembolic events

## References

[1] Amar L, Harbuz-Miller I, Turcu AF. Adrenal incidentaloma-innocent bystander or intruder? J Clin Endocrinol Metab. 2024;109(3):e1303‐e1304. 37622650 10.1210/clinem/dgad504 PMC10876404

[2] Ebbehoj A, Li D, Kaur RJ, et al Epidemiology of adrenal tumours in Olmsted County, Minnesota, USA: a population-based cohort study. Lancet Diabetes Endocrinol. 2020;8(11):894‐902. 33065059 10.1016/S2213-8587(20)30314-4 PMC7601441

[3] Herndon J, Bancos I. Diagnosing and managing adrenal incidentalomas. JAAPA. 2023;36(5):12‐18. 10.1097/01.JAA.0000923528.75127.88 37043721

[4] Sherlock M, Scarsbrook A, Abbas A, et al Adrenal incidentaloma. Endocr Rev. 2020;41(6):775‐820. 32266384 10.1210/endrev/bnaa008 PMC7431180

[5] Bancos I, Prete A. Approach to the patient with adrenal incidentaloma. J Clin Endocrinol Metab. 2021;106(11):3331‐3353. 34260734 10.1210/clinem/dgab512 PMC8530736

[6] Vassiliadi DA, Delivanis DA, Papalou O, Tsagarakis S. Approach to the patient with bilateral adrenal masses. J Clin Endocrinol Metab. 2024;109(8):2136‐2148. 38478374 10.1210/clinem/dgae164

[7] Fassnacht M, Tsagarakis S, Terzolo M, et al European Society of Endocrinology clinical practice guidelines on the management of adrenal incidentalomas, in collaboration with the European network for the study of adrenal tumors. Eur J Endocrinol. 2023;189(1):G1‐G42. 37318239 10.1093/ejendo/lvad066

[8] Sconfienza E, Tetti M, Forestiero V, Veglio F, Mulatero P, Monticone S. Prevalence of functioning adrenal incidentalomas: a systematic review and meta-analysis. J Clin Endocrinol Metab. 2023;108(7):1813‐1823. 36718682 10.1210/clinem/dgad044

[9] Kjellbom A, Lindgren O, Puvaneswaralingam S, Londahl M, Olsen H. Association between mortality and levels of autonomous cortisol secretion by adrenal incidentalomas: a cohort study. Ann Intern Med. 2021;174(8):1041‐1049. 34029490 10.7326/M20-7946

[10] Deutschbein T, Reimondo G, Dalmazi D, et al Age-dependent and sex-dependent disparity in mortality in patients with adrenal incidentalomas and autonomous cortisol secretion: an international, retrospective, cohort study. Lancet Diabetes Endocrinol. 2022;10(7):499‐508. 35533704 10.1016/S2213-8587(22)00100-0 PMC9679334

[11] Kjellbom A, Lindgren O, Danielsson M, Olsen H, Londahl M. Mortality not increased in patients with nonfunctional adrenal adenomas: a matched cohort study. J Clin Endocrinol Metab. 2023;108(8):e536‐e541. 36800277 10.1210/clinem/dgad074 PMC10348456

[12] Di Dalmazi G, Vicennati V, Rinaldi E, et al Progressively increased patterns of subclinical cortisol hypersecretion in adrenal incidentalomas differently predict major metabolic and cardiovascular outcomes: a large cross-sectional study. Eur J Endocrinol. 2012;166(4):669‐677. 22267278 10.1530/EJE-11-1039

[13] Morelli V, Reimondo G, Giordano R, et al Long-term follow-up in adrenal incidentalomas: an Italian multicenter study. J Clin Endocrinol Metab. 2014;99(3):827‐834. 24423350 10.1210/jc.2013-3527

[14] Prete A, Subramanian A, Bancos I, et al Cardiometabolic disease burden and steroid excretion in benign adrenal tumors: a cross-sectional multicenter study. Ann Intern Med. 2022;175(3):325‐334. 34978855 10.7326/M21-1737

[15] Sbardella E, Minnetti M, D'Aluisio D, et al Cardiovascular features of possible autonomous cortisol secretion in patients with adrenal incidentalomas. Eur J Endocrinol. 2018;178(5):501‐511. 29510982 10.1530/EJE-17-0986

[16] Remde H, Kranz S, Morell SM, et al Clinical course of patients with adrenal incidentalomas and cortisol autonomy: a German retrospective single center cohort study. Front Endocrinol (Lausanne). 2023;14:1123132. 37223045 10.3389/fendo.2023.1123132 PMC10200872

[17] Chen AX, Radhakutty A, Drake SM, Kiu A, Thompson CH, Burt MG. Cardiovascular risk markers in adults with adrenal incidentaloma and mild autonomous cortisol secretion. J Clin Endocrinol Metab. 2024;109(3):e1020‐e1028. 37967229 10.1210/clinem/dgad665

[18] Terzolo M, Pia A, Ali A, et al Adrenal incidentaloma: a new cause of the metabolic syndrome? J Clin Endocrinol Metab. 2002;87(3):998‐1003. 11889151 10.1210/jcem.87.3.8277

[19] Isand K, Feelders R, Brue T, et al High prevalence of venous thrombotic events in Cushing’s syndrome: data from ERCUSYN and details in relation to surgery. Eur J Endocrinol. 2024;190(1):75‐85. 38146835 10.1093/ejendo/lvad176

[20] Rabiei H, Shahbandi A, Sabahi M, Mandel M, Adada B, Borghei-Razavi H. Thrombosis in Cushing’s disease; raising the flag of concern. Neurosurg Rev. 2023;46(1):32. 36604392 10.1007/s10143-022-01941-x

[21] van der Pas R, Leebeek FW, Hofland LJ, de Herder WW, Feelders RA. Hypercoagulability in Cushing’s syndrome: prevalence, pathogenesis and treatment. Clin Endocrinol (Oxf). 2013;78(4):481‐488. 23134530 10.1111/cen.12094

[22] Isidori AM, Minnetti M, Sbardella E, Graziadio C, Grossman AB. Mechanisms in endocrinology: the spectrum of haemostatic abnormalities in glucocorticoid excess and defect. Eur J Endocrinol. 2015;173(3):R101‐R113. 25987566 10.1530/EJE-15-0308

[23] Wagner J, Langlois F, Lim DST, McCartney S, Fleseriu M. Hypercoagulability and risk of venous thromboembolic events in endogenous Cushing’s syndrome: a systematic meta-analysis. Front Endocrinol (Lausanne). 2018;9:805. 30745894 10.3389/fendo.2018.00805 PMC6360168

[24] van Haalen FM, Kaya M, Pelsma ICM, et al Current clinical practice for thromboprophylaxis management in patients with Cushing’s syndrome across reference centers of the European reference network on rare endocrine conditions (Endo-ERN). Orphanet J Rare Dis. 2022;17(1):178. 35505430 10.1186/s13023-022-02320-x PMC9062860

[25] Pofi R, Caratti G, Ray DW, Tomlinson JW. Treating the side effects of exogenous glucocorticoids; can we separate the good from the bad? Endocr Rev. 2023;44(6):975‐1011. 37253115 10.1210/endrev/bnad016 PMC10638606

[26] Limumpornpetch P, Morgan AW, Tiganescu A, et al The effect of endogenous cushing syndrome on all-cause and cause-specific mortality. J Clin Endocrinol Metab. 2022;107(8):2377‐2388. 35486378 10.1210/clinem/dgac265 PMC9282270

[27] Schernthaner-Reiter MH, Siess C, Micko A, et al Acute and life-threatening complications in cushing syndrome: prevalence, predictors, and mortality. J Clin Endocrinol Metab. 2021;106(5):e2035‐e2046. 33517433 10.1210/clinem/dgab058

[28] Pofi R, Giannetta E, Feola T, et al Sex-specific effects of daily tadalafil on diabetic heart kinetics in RECOGITO, a randomized, double-blind, placebo-controlled trial. Sci Transl Med. 2022;14(649):eabl8503. 35704597 10.1126/scitranslmed.abl8503

[29] Pofi R, Tomlinson JW. Is autonomous cortisol secretion sexually dimorphic? Lancet Diabetes Endocrinol. 2022;10(7):473‐475. 35533705 10.1016/S2213-8587(22)00110-3

[30] Kautzky-Willer A, Harreiter J, Pacini G. Sex and gender differences in risk, pathophysiology and complications of type 2 diabetes mellitus. Endocr Rev. 2016;37(3):278‐316. 27159875 10.1210/er.2015-1137 PMC4890267

[31] Bredella MA . Sex differences in body composition. Adv Exp Med Biol. 2017;1043:9‐27. 29224088 10.1007/978-3-319-70178-3_2

[32] Clayton JA, Gaugh MD. Sex as a biological variable in cardiovascular diseases: JACC focus seminar 1/7. J Am Coll Cardiol. 2022;79(14):1388‐1397. 35393021 10.1016/j.jacc.2021.10.050

[33] Mauvais-Jarvis F, Bairey Merz N, Barnes PJ, et al Sex and gender: modifiers of health, disease, and medicine. Lancet. 2020;396(10250):565‐582. 32828189 10.1016/S0140-6736(20)31561-0 PMC7440877

[34] Reincke M, Fleseriu M. Cushing syndrome: a review. JAMA. 2023;330(2):170‐181. 37432427 10.1001/jama.2023.11305

[35] Nieman LK, Biller BM, Findling JW, et al The diagnosis of Cushing’s syndrome: an Endocrine society clinical practice guideline. J Clin Endocrinol Metab. 2008;93(5):1526‐1540. 18334580 10.1210/jc.2008-0125 PMC2386281

[36] Mancia G, Kreutz R, Brunstrom M, et al 2023 ESH guidelines for the management of arterial hypertension the task force for the management of arterial hypertension of the European society of hypertension: endorsed by the international society of hypertension (ISH) and the European renal association (ERA). J Hypertens. 2023;41(12):1874‐2071. 37345492 10.1097/HJH.0000000000003480

[37] Cosentino F, Grant PJ, Aboyans V, et al 2019 ESC guidelines on diabetes, pre-diabetes, and cardiovascular diseases developed in collaboration with the EASD. Eur Heart J. 2020;41(2):255‐323. 31497854 10.1093/eurheartj/ehz486

[38] Matthews DR, Hosker JP, Rudenski AS, Naylor BA, Treacher DF, Turner RC. Homeostasis model assessment: insulin resistance and beta-cell function from fasting plasma glucose and insulin concentrations in man. Diabetologia. 1985;28(7):412‐419. 3899825 10.1007/BF00280883

[39] Khan F, Judge EP, Das JP, et al Effects of active chronic cigarette-smoke exposure on circulating fibrocytes. Lung. 2024;202(4):431‐440. 38935158 10.1007/s00408-024-00720-3 PMC11272705

[40] Delgado G, Siekmeier R, Grammer TB, Boehm BO, Marz W, Kleber ME. Alterations in the coagulation system of active smokers from the Ludwigshafen risk and cardiovascular health (LURIC) study. Adv Exp Med Biol. 2015;832:9‐14. 25300683 10.1007/5584_2014_5

[41] Cheng YJ, Liu ZH, Yao FJ, et al Current and former smoking and risk for venous thromboembolism: a systematic review and meta-analysis. PLoS Med. 2013;10(9):e1001515. 24068896 10.1371/journal.pmed.1001515 PMC3775725

[42] Pastori D, Cormaci VM, Marucci S, et al A comprehensive review of risk factors for venous thromboembolism: from epidemiology to pathophysiology. Int J Mol Sci. 2023;24(4):3169. 36834580 10.3390/ijms24043169 PMC9964264

[43] Bonaventura I, Minnetti M, Ferrari D, et al Sex and cortisol impact on coagulation profile in adrenal incidentalomas: a single-centre study-Supplemental Materials-Table S1. 2024. 10.6084/m9.figshare.26068270.v1

[44] Bonaventura I, Minnetti M, Ferrari D, et al Sex and cortisol impact on coagulation profile in adrenal incidentalomas: a single-centre study-Supplemental Materials-Figure S1. 2024. 10.6084/m9.figshare.26068267.v1

[45] Fleseriu M, Auchus R, Bancos I, et al Consensus on diagnosis and management of Cushing’s disease: a guideline update. Lancet Diabetes Endocrinol. 2021;9(12):847‐875. 34687601 10.1016/S2213-8587(21)00235-7 PMC8743006

[46] Guarnotta V, Amato MC, Pivonello R, et al The degree of urinary hypercortisolism is not correlated with the severity of Cushing’s syndrome. Endocrine. 2017;55(2):564‐572. 26965912 10.1007/s12020-016-0914-9

[47] Barzon L, Sonino N, Fallo F, Palu G, Boscaro M. Prevalence and natural history of adrenal incidentalomas. Eur J Endocrinol. 2003;149(4):273‐285. 14514341 10.1530/eje.0.1490273

[48] Barzon L, Scaroni C, Sonino N, Fallo F, Paoletta A, Boscaro M. Risk factors and long-term follow-up of adrenal incidentalomas. J Clin Endocrinol Metab. 1999;84(2):520‐526. 10022410 10.1210/jcem.84.2.5444

[49] Minnetti M, Hasenmajer V, Sbardella E, et al Susceptibility and characteristics of infections in patients with glucocorticoid excess or insufficiency: the ICARO tool. Eur J Endocrinol. 2022;187(5):719‐731. 36102827 10.1530/EJE-22-0454 PMC9641788

[50] Biino G, Santimone I, Minelli C, et al Age- and sex-related variations in platelet count in Italy: a proposal of reference ranges based on 40987 subjects’ data. PLoS One. 2013;8(1):e54289. 23382888 10.1371/journal.pone.0054289 PMC3561305

[51] Sabrkhany S, Kuijpers MJE, Van Kuijk SMJ, Griffioen AW, Oude Egbrink MGA. Age- and gender-matched controls needed for platelet-based biomarker studies. Haematologica. 2023;108(6):1667‐1670. 36200425 10.3324/haematol.2022.281726 PMC10230407

[52] Favero V, Prete A, Mangone A, et al Inflammation-based scores in benign adrenocortical tumours are linked to the degree of cortisol excess: a retrospective single-centre study. Eur J Endocrinol. 2023;189(5):517‐526. 37962923 10.1093/ejendo/lvad151

[53] Bonaventura I, Tomaselli A, Angelini F, et al Predicting postoperative hypocortisolism in patients with non-aldosterone-producing adrenocortical adenoma: a retrospective single-centre study. J Endocrinol Invest. 2024;47(7):1751‐1762. 38386266 10.1007/s40618-023-02283-1 PMC11196308

[54] Koster T, Blann AD, Briët E, Vandenbroucke JP, Rosendaal FR. Role of clotting factor VIII in effect of von Willebrand factor on occurrence of deep-vein thrombosis. Lancet. 1995;345(8943):152‐155. 7823669 10.1016/s0140-6736(95)90166-3

[55] Jenkins PV, Rawley O, Smith OP, O'Donnell JS. Elevated factor VIII levels and risk of venous thrombosis. Br J Haematol. 2012;157(6):653‐663. 22530883 10.1111/j.1365-2141.2012.09134.x

[56] Kyrle PA, Minar E, Hirschl M, et al High plasma levels of factor VIII and the risk of recurrent venous thromboembolism. N Engl J Med. 2000;343(7):457‐462. 10950667 10.1056/NEJM200008173430702

[57] Rietveld IM, Lijfering WM, le Cessie S, et al High levels of coagulation factors and venous thrombosis risk: strongest association for factor VIII and von Willebrand factor. J Thromb Haemost. 2019;17(1):99‐109. 30471183 10.1111/jth.14343

[58] Koster T, Rosendaal FR, Reitsma PH, van der Velden PA, Briët E, Vandenbroucke JP. Factor VII and fibrinogen levels as risk factors for venous thrombosis. A case-control study of plasma levels and DNA polymorphisms–the Leiden Thrombophilia Study (LETS). Thromb Haemost. 1994;71(6):719‐722. 7974338

[59] Bobbert P, Stellbaum C, Steffens D, et al Postmenopausal women have an increased maximal platelet reactivity compared to men despite dual antiplatelet therapy. Blood Coagul Fibrinolysis. 2012;23(8):723‐728. 23135379 10.1097/MBC.0b013e32835824b3

[60] Rauch U . Gender differences in anticoagulation and antithrombotic therapy. Handb Exp Pharmacol. 2012;214:523‐542. 10.1007/978-3-642-30726-3_23 23027465

[61] Lowe GD, Rumley A, Woodward M, et al Epidemiology of coagulation factors, inhibitors and activation markers: the third Glasgow MONICA survey. I. Illustrative reference ranges by age, sex and hormone use. Br J Haematol. 1997;97(4):775‐784. 9217176 10.1046/j.1365-2141.1997.1222936.x

[62] Caulin-Glaser T, Farrell WJ, Pfau SE, et al Modulation of circulating cellular adhesion molecules in postmenopausal women with coronary artery disease. J Am Coll Cardiol. 1998;31(7):1555‐1560. 9626834 10.1016/s0735-1097(98)00145-4

[63] Hernaez A, Lassale C, Castro-Barquero S, et al Mediterranean diet maintained platelet count within a healthy range and decreased thrombocytopenia-related mortality risk: a randomized controlled trial. Nutrients. 2021;13(2):559. 33567733 10.3390/nu13020559 PMC7915168

[64] De Nucci S, Bonfiglio C, Donvito R, et al Effects of an eight week very low-calorie ketogenic diet (VLCKD) on white blood cell and platelet counts in relation to metabolic dysfunction-associated steatotic liver disease (MASLD) in subjects with overweight and obesity. Nutrients. 2023;15(20):4468. 37892542 10.3390/nu15204468 PMC10610501

[65] McEwen BJ . The influence of diet and nutrients on platelet function. Semin Thromb Hemost. 2014;40(2):214‐226. 24497119 10.1055/s-0034-1365839

[66] Vogg N, Kurlbaum M, Deutschbein T, Grasl B, Fassnacht M, Kroiss M. Method-specific cortisol and dexamethasone thresholds increase clinical specificity of the dexamethasone suppression test for cushing syndrome. Clin Chem. 2021;67(7):998‐1007. 33997885 10.1093/clinchem/hvab056

[67] Pandor A, Tonkins M, Goodacre S, et al Risk assessment models for venous thromboembolism in hospitalised adult patients: a systematic review. BMJ Open. 2021;11(7):e045672. 10.1136/bmjopen-2020-045672 PMC8323381 34326045

